# Skin Collagen Fiber Molecular Order: A Pattern of Distributional Fiber Orientation as Assessed by Optical Anisotropy and Image Analysis

**DOI:** 10.1371/journal.pone.0054724

**Published:** 2013-01-18

**Authors:** Juliana Fulan Ribeiro, Eli Heber Martins dos Anjos, Maria Luiza S. Mello, Benedicto de Campos Vidal

**Affiliations:** Department of Structural and Functional Biology, Institute of Biology, University of Campinas (Unicamp), Campinas, São Paulo, Brazil; University Hospital Hamburg-Eppendorf, Germany

## Abstract

**Background:**

Birefringence can reveal much of the morphology, molecular order, heterogeneity of fiber orientation, and nonlinear optical properties of biopolymers such as collagen. However, the detailed characterization of skin collagen fibers using optical anisotropy methods remains elusive. A clear understanding of collagen fiber organization in skin tissues may be important in the interpretation of their structural-functional relationships under normal and pathological conditions. In this study, fiber orientation in collagen bundles (CBs) and their supramolecular organization were examined in rat skin using polarization microscopy and image analysis.

**Methodology/Principal Findings:**

Image variations with rotation of the microscope stage and selection of the in-depth focus plane were investigated in unstained sections of varying thicknesses from rat skin fragments. Total birefringence (image analysis) and form and intrinsic birefringence (Sénarmont’s method) were estimated. Based on the birefringent images, CBs were found to contain intercrossing points with a twisted helical distribution of collagen fibers (chiral elements) and frequently presented circular structures. Collagen fibers were observed to extend from the surface level to deeper planes, creating a 3D-network of oriented intertwined CBs. At least three levels of birefringent brilliance intensity were revealed by image analysis, indicating a heterogeneous spatial organization of the CBs. Slight differences in optical retardations were found for CBs immersed in some of the fluids used in a comparison of 170- and 240-day old rats.

**Conclusion/Significance:**

Polarization microscopy studies provide detailed high-quality structural information on rat skin CBs. A 3D-network structure based on image analysis and birefringence compensation for collagen fibers is suggested for CBs. Form and intrinsic birefringence evaluation can reveal differences in the rat skin associated with age at the levels of collagen fiber crystallinity and macromolecular organization. These findings may inspire future studies of the feedback mechanisms by which spatial, bioelectrical and biomechanical information is transmitted from CBs to skin cells.

## Introduction

In the skin, collagen fibers form higher-order networks that constitute the major dermal structure responsible for the skin’s biomechanical properties. Analysis of the variation in distribution, or morphological anisotropy, of collagen fibers is necessary to fully understand the structure-function relationship of skin tissues under normal and pathological conditions. In the context of the micro-architecture of dermal collagen and the skin’s biomechanical properties, it has been proposed that collagen fibers form “an arrangement which allows continual movements of the individual fibres to absorb the minor stresses of normal activity and relies on the ultimate strength of collagen to resist severe stretch” [1]. An intertwined meshwork of collagen fibers exists in the dermis such that “in whatever direction it is stretched, all the fibers eventually become parallel” [1]. By using polarization-sensitive optical coherence tomography (PS-OCT) to study anisotropic properties in collagen fibers of the dermis, it has been reported that when the skin is subjected to tension, rope-like structures appear in a direction parallel to the direction of the applied tension [2]. This finding agrees with the detailed observations made by Gibson et al [1].

PS-OCT is a non-invasive imaging technique that, along with extensive mathematical studies, has important clinical applications [3] and that even without mathematical manipulation has permitted the assessment of tissue microstructure [4]. The use of PS-OCT for collagen studies, including those conducted in skin tissues is based on the fact that collagen fibers exhibit optical anisotropic properties such as birefringence. PS-OCT in skin generates back-scattered light that allows the detection of collagen fiber molecular orientation and creates images amenable to measurement [2], [5]–[7].

As collagen fibers have a heterogeneous distribution in the skin, authors commonly refer to this organizational state as anisotropy of distribution, or “directional variations in tissue organization” [8]. To avoid confusion, it is necessary to differentiate this type of anisotropy from optical anisotropy, which identifies intrinsic and form birefringence and linear dichroism, physical properties exhibited by collagen molecule-forming fibers. Studies on the optical anisotropy of collagen fibers organized as bundles (CBs) are possible because of the molecular and supramolecular orientation of their biopolymer components.

Research on fiber orientation in CBs is important not only for human clinical applications but also for industrial and cosmetological uses. The strength and softness of skin and leather are dependent on the supra-organization of their collagen fibers [9], [10]. Optical anisotropy properties as birefringence can reveal much of the morphology, molecular order, heterogeneity of fiber orientation and nonlinear optical properties in biopolymers such as collagen.

Two types of birefringence have been reported for collagen fibers, particularly those in tendons and cartilage: intrinsic or crystalline birefringence (B_i_) and form birefringence (B_f_). B_i_ is caused by the orientations and the oscillatory strengths of all electronic transitions of the molecules within an oriented structure. The formula for the B_i_ value is given by: B_i_ = n_e_ – n_o_, where n_e_ = the extraordinary ray refractive index relative to the direction parallel to the major axis of the collagen fiber, and n_o_ = the ordinary ray refractive index relative to the direction perpendicular to the major axis of the collagen fiber. B_f_ is caused by the dispersion with a preferential orientation of asymmetrical particles of a given refractive index in a medium of a different refractive index. B_f_ is the anisotropy of the dielectric constant for a given frequency and can also be considered a nonlinear optical property [11]–[15]. In the case of collagen fibers, B_f_ arises as a function of the geometry and orientation of the rod-shaped triple chain collagen molecule (2,800 Å long and approximately 15 Å thick) and the packing degree of these fibers. B_i_ results from the asymmetrical alignment of chemical bonds or ions within collagen particles and is independent of the refractive index of the immersion fluid [11].

As measurable properties, B_i_ and B_f_ have been used for the comparison of CBs in tendons, cartilages and cornea, revealing information of vital importance to the detection of changes in the macromolecular organization of CBs under different physiological and pathological conditions [15 - review]. Data on B_i_ and B_f_ have informed changes in the structural organization of collagen fibers during the process of tendon repair after surgical removal [16] and the increased packing state of collagen fibers with aging in the Achilles tendon [17]. During the process of ossification, changes in collagen type II-rich orientation are revealed by the analysis of form birefringence curves [18], [19]. Bi and Bf studies have also provided information on the following topics: the decrease of molecular organization in collagen observed in the floppy valve disease [20]; the increase in the molecular orientation of CBs in tendons during exercise [21]; the beneficial effect of therapeutic ultrasound on the organization and aggregation of CBs in Achilles tendons during the healing process [22]; the degree of crystallinity and the spatial orientation of collagen fibers in the porcine cornea [23] and changes in those same properties in the corneas of diabetic mice [24]; and differences in the oriented arrangement of collagen type I-rich structures when comparing chordae tendineae to tendons in pigs [25]. However, no such approach has been used to study CBs in the skin and to characterize their optical properties in detail.

In this work, we studied birefringence images of collagen bundles in rat skin using polarization microscopy and image analysis to obtain a better understanding of their supramolecular organization. Images of birefringence were also analyzed in thick sample sections to gain insight into the in-depth orientations of collagen in the skin.

## Results

Birefringent CB images were observed in rat skin sections cut at varying thicknesses. The birefringence intensity was found to vary as a function of the fiber orientation as related to crossed polarizers and as a function of the section thickness.

**Figure 1:**
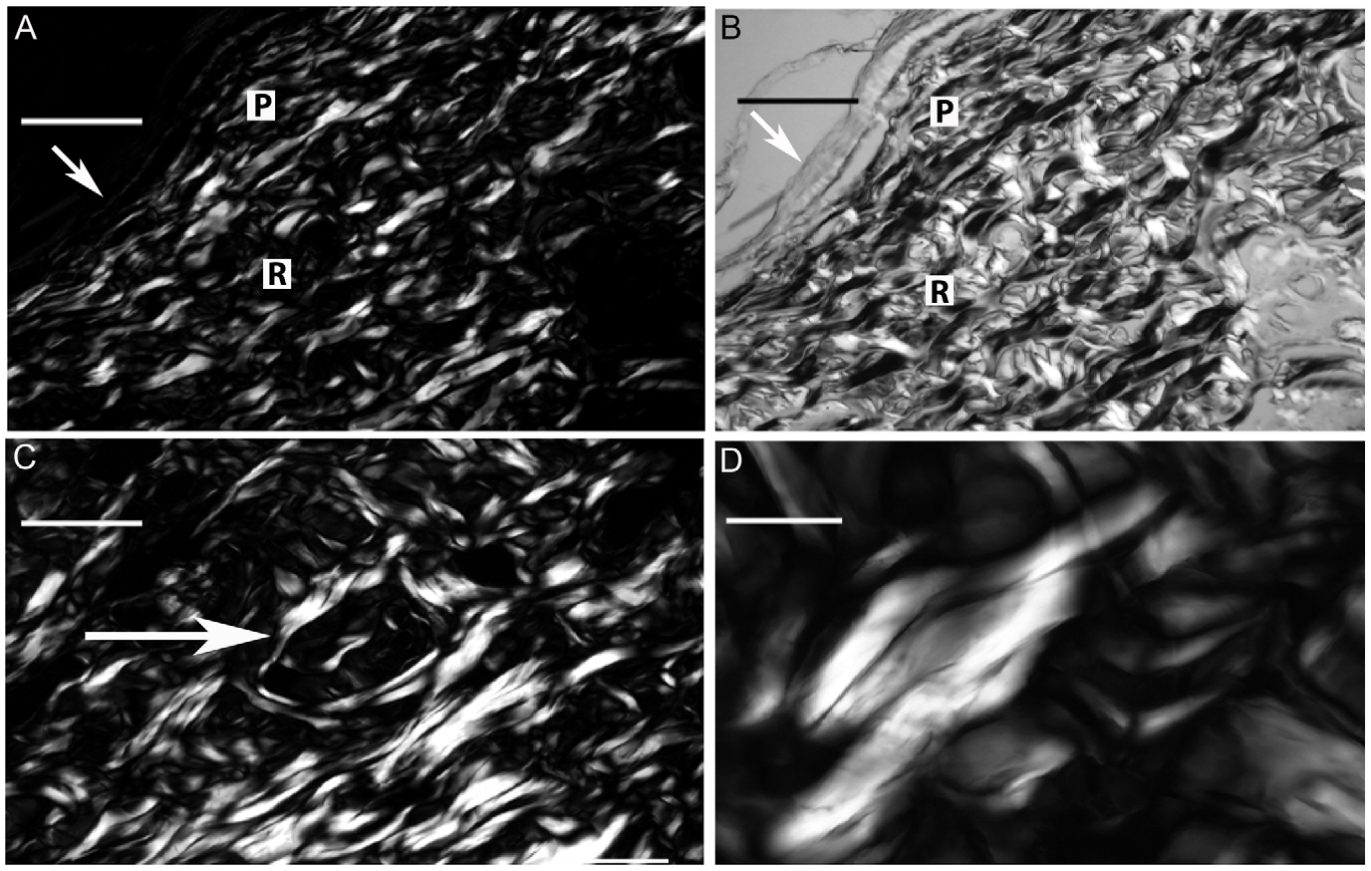
Birefringent images of skin CBs. In the papillary layer (P), CBs are distributed parallel to the epithelial surface (arrow) and are thinner than the CBs of the reticular region (R), which are orthogonally distributed. The birefringence brightness originally exhibited by the CBs in the papillary layer in A appears compensated for in black after using Sénarmont’s method in B, provided that these structures are positioned at 45° with respect to the crossed polarizer-analyzer planes. The non-compensated birefringence in B is evident for CBs positioned perpendicularly to the CBs exhibiting birefringence compensation. CBs with helically intertwined and chiral aspects forming circular structures in the reticular region can be identified due to their birefringence characteristics (C, arrow). Different brilliances and hues of gray are also observed in the birefringent images of the CBs depending on their patterns of macromolecular orientation in the dermis (D). Bars = 100 µm (A–C) and 50 µm (D).

**Figure 2:**
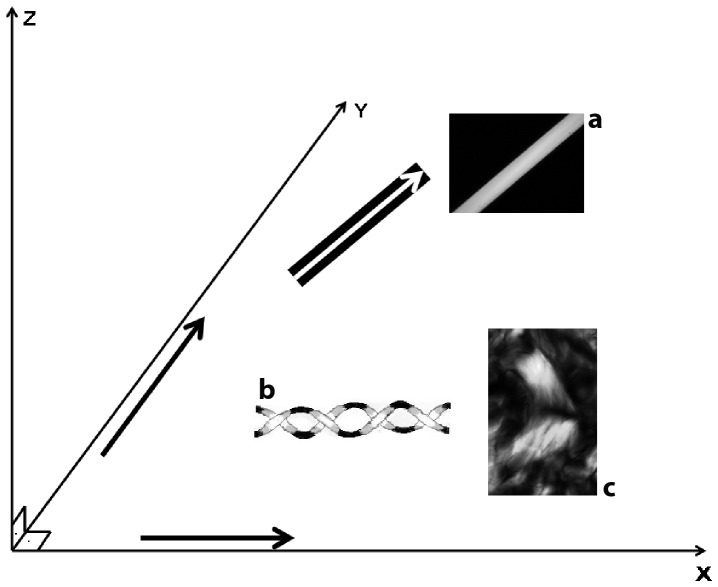
Schematic representation and images of birefringence brilliance meaning. Representation of the oriented relationship of collagen fibers or CBs with respect to the plane of polarized light (PPL) of the microscope polarizer (X-axis) and analyzer (Y-axis). During examination of the samples, the microscope polarizer and the analyzer remain crossed, while the microscope stage is rotated to detect changes in the birefringence brilliance of the CBs relative to their orientation. The Z-axis represents birefringence brilliance variance in OR, which is represented as 3D information because the birefringence brilliance intensity depends on the CB orientation with respect to the PPL of the crossed polarizer-analyzer [15], [23]. High OR values are obtained in the Z-axis when the long axis of a fiber (for example, a 14.8-µm-thick nylon fiber with maximal internal molecular order) (a) is oriented at 45° (the direction of the white arrow on the black background) with respect to the crossed polarizer-analyzer (X and Y). When the long axis of this fiber is oriented parallel to one of the crossed polarizers (black arrows) by rotation of the microscope stage, birefringence extinction occurs. A CB fiber hypothetically composed of two helical chains is represented (b), such that when the long axis of the fiber is positioned parallel to the polarizers, only the portion of the chains parallel to the polarizer will appear black, whereas the portion of the chains positioned at 45° with respect to the crossed polarizer-analyzer will show birefringence brilliance. Differently oriented collagen birefringent images are also shown (c).

In sections immersed in water for general observation of birefringence, CBs were observed in orientations nearly perpendicular to each other (Fig. 1A). This pattern was better detected and understood using Sénarmont’s compensation method, through which CBs with compensated birefringence appear dark while non-compensated CB birefringence increases in brilliance intensity (Fig. 1B). The birefringence brilliance varies as a function of the angle of fiber orientation in the CBs (see schematic representation in Fig. 2).

Each twisted CB was shown to be part of a larger circular structure (Fig. 1C). The CB groups varied in birefringence brilliance (Fig. 1D), as a consequence of variation in the collagen fiber path in individual CBs and the trajectory of the CB path variation within the section depth.

These observations were independent from the ages of rats used.

**Figure 3:**
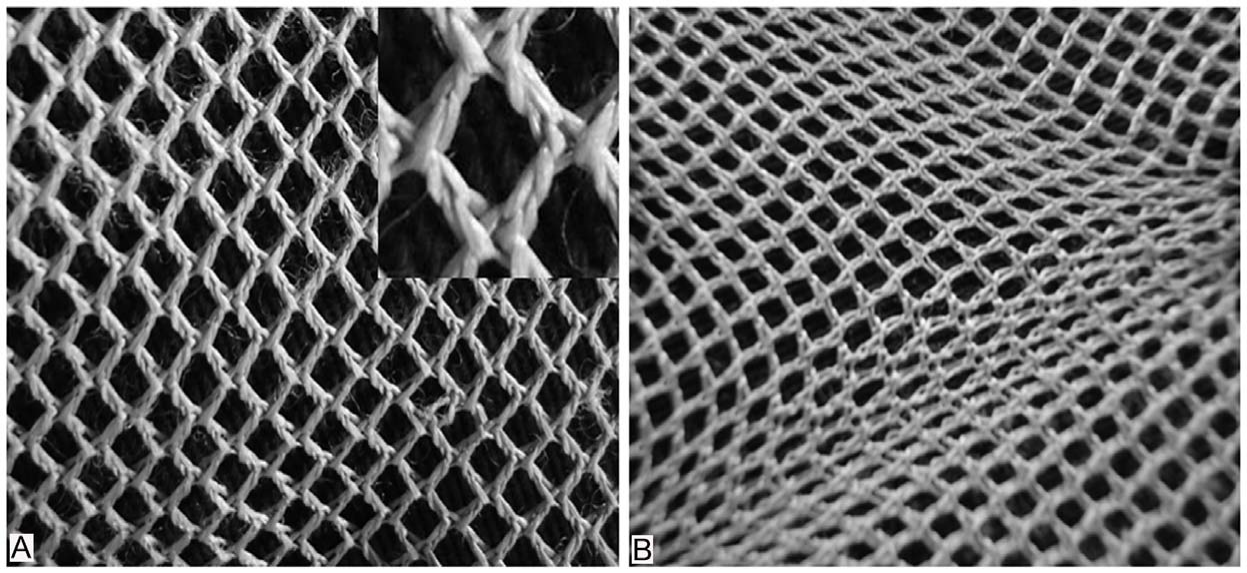
A model of collagen organization in skin. An interpretation of how a collagen fiber network is interwoven (A), based on work from Gibson et al. [1– Fig. 12]. The insert in A is a higher magnification of the intertwined fibers, also showing a 3D distribution of the fibers. When this network is stretched in any direction, the fibers become oriented parallel to the stretching direction (B).

Based on reported findings obtained with a different methodology [1] and on the birefringence images depicted in Figures 1A-B of the present study, a 2D-pattern for the distribution of CBs in the skin is proposed here (Fig. 3A,B). The insert in Figure 3 shows, in detail, the twisting and entanglement polymer fibers representing CB fibers, in which a 3D aspect is even perceived.

### Rotating the Stage of the Polarizing Microscope Yields Image Variations

To detect the true orientation of the CBs and of the fibers inside CBs, it was necessary to gradually rotate the stage of the polarizing microscope while maintaining the field of observation constant. This rotation was performed to verify whether variation of the CB axis occurred with respect to the polarizer and analyzer and to compensate birefringence with the use of the Sénarmont’s compensator.

**Figure 4:**
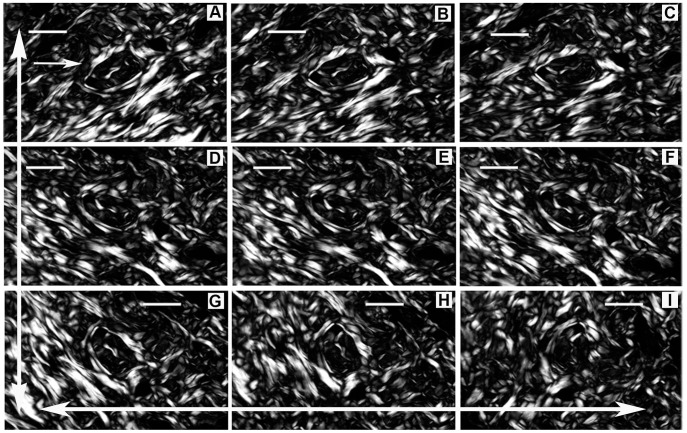
Birefringence of skin CBs observed after rotating the stage of the polarizing microscope. Although birefringence as a phenomenon is evident in CBs in any given position of the field of observation while rotating the microscope stage from A to I, changes in the birefringent image characteristics are observed. A. Most CBs are positioned at 45° with respect to the polarizers; a circular arrangement of CBs is evident in the center of this image. Such an arrangement is maintained in B through I, despite changes in their birefringence. D. Most CBs appear to be oriented at 45° with respect to the polarizer-analyzer, but they are oriented in the opposite direction with respect to the preceding figures. Following the clockwise rotation of the microscope stage from D-I, changes in the birefringent images continue to be observed. I. Almost all fibers forming the CBs attain a relatively maximal extinction position. The large arrows in the X- and Y-axis directions represent the polarizer and analyzer PPL, respectively. Bar = 100 µm.

When the microscope stage was rotated clockwise, differences in birefringence brilliance could be perceived in the organized distribution of the collagen fibers in CBs which presumes that these fibers could acquire a variable orientation within the 3D space. This is shown in nine of the most informative images, which displayed changes in birefringence relative to the angle of the CB axis with respect to the plane of polarized light of the polarizer-analyzer (Fig. 4). Observations began with the image exhibiting the greatest intensity of birefringence brilliance, which occurred when the collagen fibers’ axes were oriented at 45^o^ with respect to the crossed polarizers from the southwest to the northeast direction (Fig. 4A). Following the clockwise rotation of the microscope stage caused a decrease in the birefringence brilliance of these fibers that was related to the decreased angle of the fibers’ axes with respect to the plane of polarized light (Fig. 4B). When the fibers’ axes were aligned parallel to the polarizer’s plane of polarized light, there was extinction of the birefringence brilliance; consequently, fibers which appeared birefringent in Fig. 4A became entirely black (Fig. 4C). From 4A to 4B, changes in the birefringence brilliance of the collagen fibers were observed as a function of changes in their relative angles with respect to the crossed polarizers. Notably, some birefringent images were not detected because some of the collagen fibers’ axes were positioned parallel to one of the polarizers’ planes of polarized light (extinction position) (Fig. 2). After rotating the microscope stage to the appropriate 45^o^ angle, these images appeared brilliant (Fig. 4). The changes in the birefringent CB images delimit a circular structure containing a twisted component, which can be seen in the images at the center of Figure 4.

From Figure 4D to 4F, a successive radical change occurred relative to the angle of the CB fibers’ orientation. The CBs were oriented from the southeast to the northwest. Changes in the birefringence brilliance were then detected, obeying the rule of maximal birefringence brilliance occurrence when the long axes of the fibers were positioned at 45^o^ with respect to the crossed polarizers. The extinction position occurred when the long axes of the fibers were parallel to the plane of polarized light of either the polarizer or the analyzer (Fig. 2). Continuing the rotation of the microscope stage (Fig. 4G–I) led to the appearance of different birefringent images, in particular in those CBs that formed circular structures. At any angle formed between the CB fiber axes and the plane of polarized light, birefringence was displayed as a result of the CB interweaving/intercrossing patterns. Fibers or portions of fibers oriented at or near 45^o^ to the plane of polarized light and exhibiting various hues from brilliance to darkness were always observed.

These observations were independent from the ages of rats used.

### Birefringence Variation with Varying Microscope Focal Planes

**Figure 5:**
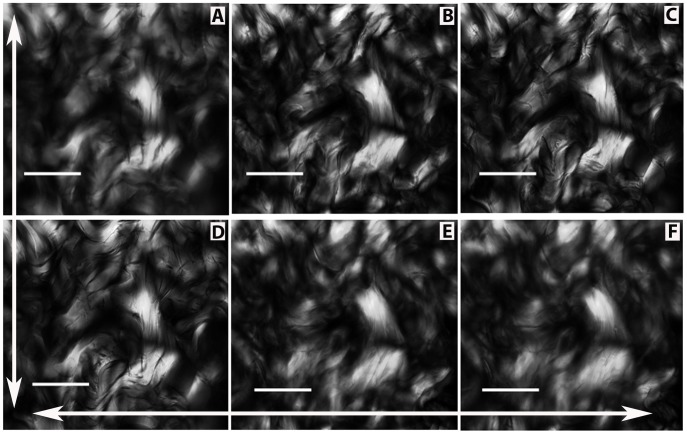
Birefringent images with focal plane changes. The microscope’s focal plane was sequentially altered from the upper to the lower surface (A to F) of a thick section of skin. The morphological changes observed depend on the track followed by the CBs from one focal position to the next. The large arrows in the X- and Y-axis directions represent the polarizer and analyzer PPL, respectively. Bar = 50 µm.

**Figure 6:**
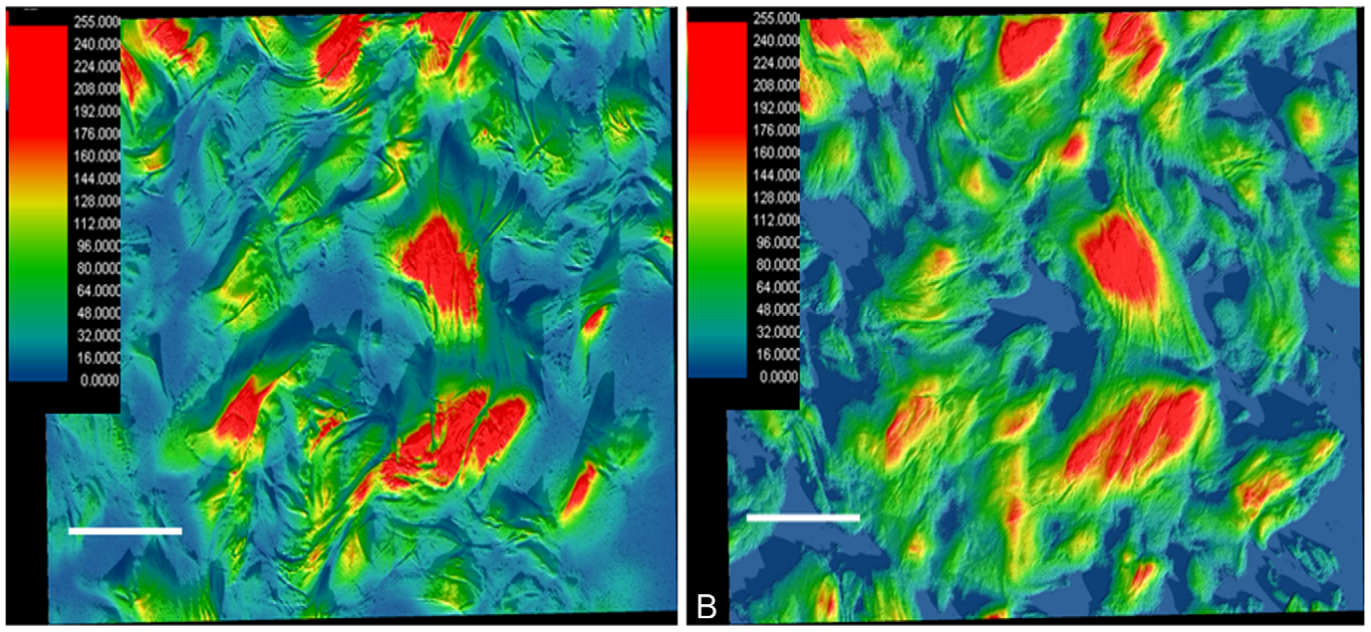
Surface plots of birefringent images of skin CBs. In A and B, false colors were correlated with the birefringence brilliance images shown in Figures 5A and 5F, respectively. The differences in image focal planes due to the paths followed by the CBs from one focal plane to the subsequent deeper one in the same section are represented by different colors. The scale inserted on the left in A and B corresponds with pixel values that are representative of the various false colors. Bar = 50 µm.

Differences in birefringence intensities could be obtained by varying the microscope focal plane in thick skin sections. Comparison of the most superficial layer of the section (Fig. 5A) to deeper focal layers (Fig. 5B–F) revealed that collagen fibers through multiple levels of the section, creating 3D network of intertwined CBs. This information can be validated using the Surface Plot item from the Measurement Table of Image-ProPlus version 6.3 software, such that Figure 6A matches Figure 5A and Figure 6B corresponds to Figure 5F. The resulting images illustrate the changes in the CB path that are detected by changing the microscope focal plane. The different colors observed are directly proportional to the gray values of the original images.

**Figure 7:**
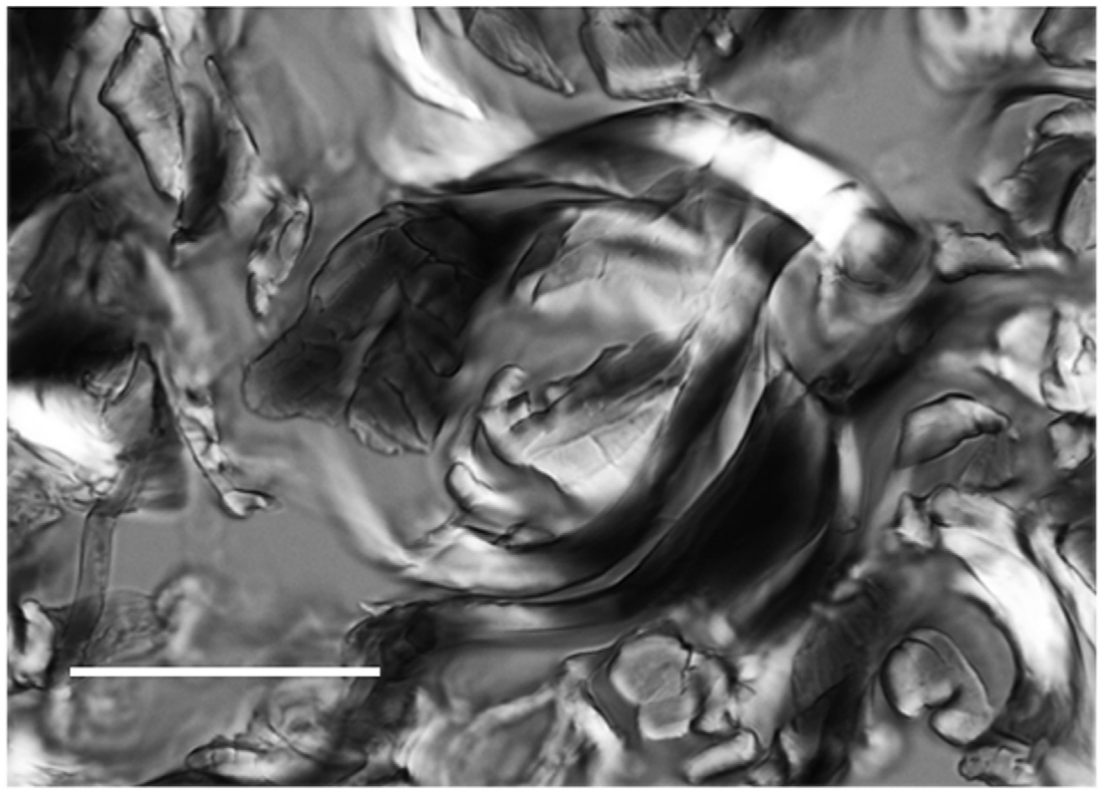
Circular and helical arrangement aspects of skin CBs. A 3D circular arrangement of CBs is envisioned in a thick skin section. Birefringence compensation is observed in the collagen fibers oriented at 45° with respect to the crossed polarizer-analyzer. The various hues of gray are a function of differences in fiber orientation, resulting in birefringent images that range from complete compensation (black) to non-compensation (brilliance). Bar = 50 µm.

Analysis of the birefringent image at a single focal plane in the ∼40-µm-thick skin section displays crossing points in the CBs that constitute an intercrossing node delimiting a circular structure (Fig. 7). A 3D view was then perceived after employing Sénarmont’s compensation, which results in the observation of black interwoven fibers and non-compensated birefringent brightness in the CBs (Fig. 7). These findings did not vary with the age of rats used.

### Birefringence Measurements Obtained by Image Analysis

**Figure 8:**
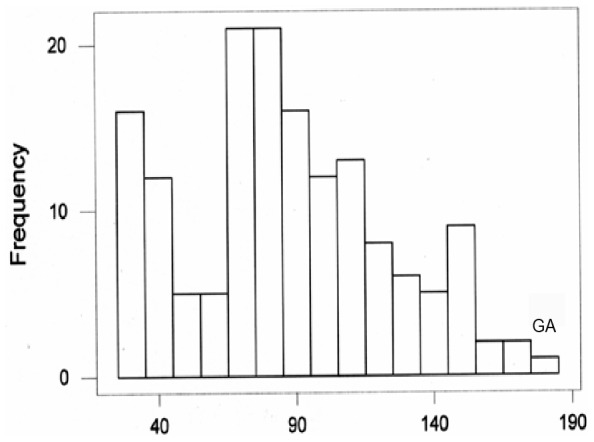
Birefringence image analysis of skin CBs. A frequency histogram of birefringence gray average (GA) values expressed in pixels, obtained after the skin sections were immersed in water and the long axes of the skin CBs were positioned at 45° with respect to the crossed polarizer-analyzer. A polydispersed distribution of the GA values is observed.

The birefringence brilliance of CBs in 8-µm-thick skin sections immersed in distilled water, measured as gray values in pixels, allowed the software to calculate values for the gray average (GA) for each of the 154 areas that composed the total measured area of 164,733 µm^2^. A polymodal distribution of GA values comprising at least three levels of brilliance intensity was revealed for the GA values that were plotted as a frequency histogram for 240-day-old rats (Fig. 8). The arithmetic mean and its standard deviation for all GA values were found to be 124.5 and 70.4 pixels, respectively, reflecting the inhomogeneity of birefringence brilliance.

### B_f_ and B_i_ Measurements Obtained Using Sénarmont’s Method, (1/4 λ Compensator)

**Figure 9:**
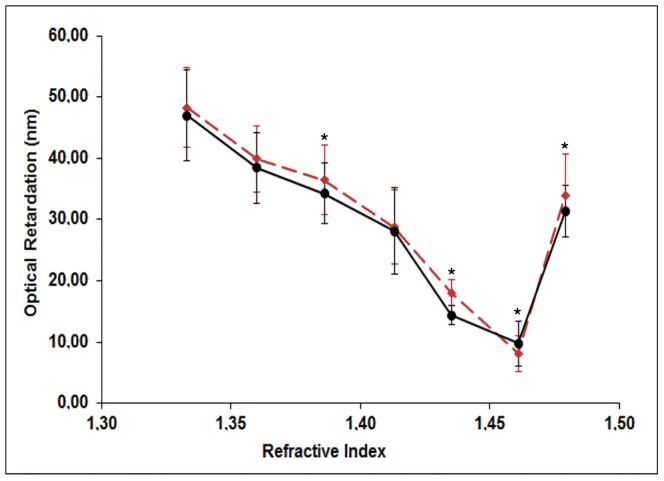
Skin CB birefringence varies as a function of the refractive index of the immersion fluids. Plots of optical retardation (OR) values in nanometers against the refractive index of the different immersion fluids were obtained for the skin CBs in 170-day-old (red line) and 240-day-old (black line) rats. Each point on the curves is the arithmetic mean of 90 OR values from four rats. Error bars are indicated. Asterisks indicate differences significant at P_<0.05_, as determined by ANOVA, when comparing OR values obtained from the skin of rats of different ages. B_i_ is identified at the refractive index (1.46) corresponding to the smallest OR obtained. See text for details.

B_f_ and B_i_ can be found by examining the form birefringence curves for CBs in skin from 170-day-old and 240-day-old rats (Fig. 9). The smallest mean optical retardation (OR) values, which correspond to B_i_, were found for both ages at a refractive index of 1.46. The highest OR values, mainly contributed by B_f_, were also found in both cases at n of 1.33. At some refractive indices, including that corresponding to B_i_, the OR values obtained were slightly affected by age (Fig. 9). The B_i_ OR values for CBs from 240-day-old rats were found to increase in comparison with those for CBs from 170-day-old rats (Fig. 9).

## Discussion

The birefringent characteristics of CBs detected in rat skin reveal that their supramolecular organization can be accurately studied in histological sections examined with polarization microscopy and image analysis. The skin CBs consist of birefringent fibers that change their paths as the overall CB orientation twists. The details of collagen fiber supraorganization and the macromolecular order (as it relates to CB formation) were found using the above-cited methodology. Based on birefringent images, an intercrossing, twisting helical distribution of collagen fibers within CBs in rat skin was demonstrated in the present work.

Although a 2D distribution model for skin CBs was proposed in Figure 3, the observations made in 40-µm-thick skin sections by varying the microscope’s plane of focus from the surface down to deeper section planes allowed for an expanded 3D pattern visualization of CB spatial distribution. As the birefringent image of the CBs varied with the orientation of the CB axis with respect to the plane of polarized light of the crossed polarizers, we concluded that CBs are variably oriented in rat skin.

The discovery of birefringent circular CB structures in the rat dermis differs from the observation of ring-shaped structures surrounding the infundibula in human skin follicles [2]. In the present study, the circular structures were detected far from the follicle region and exhibited different morphology.

The quality of the birefringent images and the reproducibility of the birefringence results obtained with the advanced polarization microscopy used herein improve upon those obtained by PS-OCT [2], [4]–[7]. Despite the clinical advantages provided by PS-OCT [2], [4]–[7], the technique may not be capable of detecting birefringence changes in deep regions of the skin [7]. It is important to consider that collagen optical anisotropies demonstrated through advanced polarized light microscopy, such as that used in the present study, furnish relevant and quantitative structural details of the orientation patterns of CBs in the reticular region of the skin.

The polydispersed distribution of GA values identified by image analysis and associated with CB birefringence brilliance in the skin reveals the high variability in the spatial orientation of CBs in this tissue.

Analysis of the form birefringence curves for rat skin CBs using Sénarmont’s method identified B_f_ and B_i_ birefringence types. B_f_ is an optical non-linear property [13], [14] to which Bêche and Gaviot’s [26] definition for two components applies. In the context of muscle form birefringence, other investigators have reported that “in the case of muscle the principal constituent dielectrics are the thick and thin filaments and the sarcoplasm in which they are immersed. The dielectric constant (or polarizability, or refractive index) of these protein filaments is greater than that of sarcoplasm.” [27]. A similar conclusion is assumed to apply to skin CBs. Nanometric collagen molecules in collagen fibers have a higher refractive index than non-collagenic glycoproteins and oriented proteoglycans.

The fact that some differences in skin CB birefringence were found as a function of the age of the animals used may be a consequence of the age affecting CB aggregation state. In CBs from other tissues, such as rat Achilles tendons, the most significant changes in form birefringence curves with aging have been reported to occur at an age of approximately one year [17]. However, changes in CB composition and aggregation in skin may occur earlier. In tendons and cartilage, proteoglycanic components of the CB have been found to play a role in the form and intrinsic birefringence of the CB, thus affecting the profile of their form birefringence curves [15], [17], [19], [28]–[30]. The glycanic chains of proteoglycans may appear statistically to be oriented parallel to the collagen molecules, optimizing the electrostatic interactions among these molecules [27]–[29]; changes in their composition, quantity and orientation would thus be reflected in OR values and in the form birefringence curve profile [15], [17], [19]. It is likely that a similar phenomenon occurs in the skin. Indeed, age-related differences in proteoglycan composition and catabolism have been reported in human skin [31]. Analysis of B_i_ and B_f_ in skin CBs from rats under variable older rats and of the glycanic components in these CBs is a matter for future investigation.

CBs in the skin respond to stretching forces, including pressure, in almost all directions. The concept that “tendons are twisted grain boundary liquid crystals endowed with Second Harmonic Generation and B_f_” [32], [33] may be extended to skin CBs because, although differently organized in 3D-space, skin and tendon CBs have similar compositions and anisotropic optical properties. Both B_i_ identified in rat skin sections immersed in pure glycerin (n = 1.46) and elevated OR values attributed to B_f_, identified in sections immersed in water (n = 1.33), have been reported in adult tendons [17]. CB properties that give rise to higher molecular order and chirality result from physicochemical laws that affirm that a chiral molecule produces a chiral body [34]. Our results, as well as currently available information, support the proposition that collagen fibers can be a source of spatial information and of biomechanical feedback for cells [25], [32].

The findings of this study may have broader implications for improvements to applications in plastic and reconstructive surgery and skin physiotherapy.

### Conclusions

Rat skin CBs exhibit birefringence that can be quantitatively evaluated through the estimation of brilliance gray averages by image analysis and by the determination of optical retardation values using Sénarmont’s method.

Because the birefringence of collagen fibers organized in CBs revealed at least three levels of brilliance intensity in extensive section areas, as assessed by image analysis, variable CB organization can be demonstrated in rat skin.

Form and intrinsic birefringence types can be demonstrated in skin CBs by analysis of their form birefringence curves. A slight increase in OR values corresponding to B_f_ and other OR changes after CB immersion in certain fluids that occurred in the skin of 270-day-old rats, compared with 170-day-old rats, suggests that gradual changes in collagen fiber aggregation and/or proteoglycan composition may occur in rat skin CBs with advancing age, starting from an age as early as 270 days.

A patterned distribution of CBs in the skin is characterized by weaving that builds a 2D meshwork in the section plane. However, CB structures display twisted helical fibers (chiral elements) that frequently build circular structures that can extend from surface planes to deeper planes when examined in thick skin sections. Consequently, there is evidence to suggest that skin CBs form a 3D network. The CB characteristics described here inform and necessitate future studies on the feedback mechanisms by which CBs transmit spatial, bioelectrical and biochemical information to skin cells.

## Materials and Methods

### Rat Skin Sample Processing

Wistar rats aged 170 and 240 days, five from each age group, were used. The animal care protocol was approved by the Unicamp Institutional Committee for Ethics in Animal Experimentation and was in accordance with the Guidelines of the Canadian Council on Animal Care (protocol no. 2700-1). After the animals were killed in a carbonic gas chamber, they were shaved, and skin samples were collected from their medial dorsal areas.

The samples were fixed in 4% paraformaldehyde in 0.1 M phosphate buffer at pH 7.4 for 3 h under vacuum and subsequently stored for 24 h in the refrigerator. Then, the samples were rinsed for 24 h in distilled water and processed for embedding in Histosec® without DMSO (Merck, Darmstadt, Germany) at a 56–58°C melting point. No effect of paraffin embedding on the optical anisotropy of CBs has been found in tendons [35].

Unstained sections cut from the samples at 8-µm or 40-µm thicknesses were used to investigate the birefringent morphology and birefringence intensity of the samples. After being dewaxed and hydrated, the sections were immersed in distilled water and in a series of immersion media with different refractive indices to evaluate birefringence brilliance.

### Polarization Microscopy

Examination of the samples and measurements taken of CBs in the skin were performed with an Olympus BX51-P BX2 polarization microscope. Measurements were taken of CBs in the reticular region of the skin.

Birefringence characteristics were evaluated using two different procedures:

- Image analysis of the birefringence brightness, captured with a Q-Color 3 camera (Olympus America Inc., Center Valley, PA USA), after orientation of CB groups with their long axes positioned preferentially at 45° with respect to the crossed polarizer-analyzer. In this position some CBs exhibited bright birefringence, while others (those with different orientations with respect to the crossed polarizer-analyzer), appeared with various hues from bright to black due to the various characteristics of the morphological anisotropy of the skin (see Fig. 2 for details). Image-ProPlus version 6.3, using the guide for Windows™ (Media Cybernetics, Inc., Bethesda, USA), was used for image analysis of the birefringence brilliance. This procedure allowed visualization of the CBs’ paths and 3D aspects. Quantitatively, the birefringence brilliance of CBs in sections immersed in distilled water, as determined through image analysis, can be expressed as gray values in pixels. Gray values were evaluated in 154 different areas selected from the reticular regions of two different skin sections from five rats each. A gray average (GA) value was then calculated by the software as an average of the gray values established for each of the 154 areas (total area measured = 164,733 µm^2^) [36]. The GA values thereby obtained were distributed in a frequency histogram.
- Determination of birefringence optical retardations (OR) ( = optical path differences) using Sénarmont’s method. OR = (n_e_ – n_o_)t, where t = thickness of the section. This procedure was performed with the Olympus polarizing microscope using a Sénarmont’s 1/4 λ compensator and monochromatic light (λ = 546 nm) obtained with a narrow band pass interference filter (Edmund Industrial Optics, Barrington, USA). Because collagen fibers organized in bundles (CBs) are complex composites, the best way to evaluate the contribution of their B_i_ and B_f_ birefringence types was to construct curves representing changes in birefringence OR as a function of the refractive index of the immersion fluids (“form birefringence curves” [11]). Skin sections, cut 8-µm thick, were immersed in a series of fluids with increasing refractive indices (distilled water, n = 1.33; 20%, 40%, 60% and 80% glycerin-water solutions, n = 1.36–1.44; pure glycerin, n = 1.46; and nujol mineral oil, n = 1.48) [16], [29], [30], and OR values (expressed in nanometers) were evaluated under these conditions. After plotting OR values against refractive indices, the minimal OR value observed in the form birefringence curve corresponded to B_i_ [11].

### Statistical Analysis

Calculations and statistical analysis were performed with Minitab 12™ software (State College, PA, USA). ANOVA was used to assess the statistical significance for comparison of OR values. P_<0.05_ was considered the critical level for rejection of the null hypothesis.
